# Coronavirus disease 2019 (COVID-19) in pregnancy: 2 case reports on maternal and neonatal outcomes in Yichang city, Hubei Province, China

**DOI:** 10.1097/MD.0000000000021334

**Published:** 2020-07-17

**Authors:** Tingting Zheng, Jianqiang Guo, Wencong He, Hao Wang, Huiling Yu, Hong Ye

**Affiliations:** a Department of Obstetrics and Gynecology; b Emergency Department, The First College of Clinical Medical Science, Yichang Central People's Hospital, Three Gorges University, Yichang, Hubei, PR China.

**Keywords:** coronavirus disease 2019, neonate, pregnancy, vertical transmission

## Abstract

**Rationale:**

The outbreak of coronavirus disease 2019 (COVID-19) in 2019 has become a global pandemic. It is not known whether the disease is associated with a higher risk of infection in pregnant women or whether intrauterine vertical transmission can occur. We report 2 cases of pregnant women diagnosed with COVID-19.

**Patient concerns:**

In all of Yichang city from January 20, 2020, to April 9, 2020, only 2 pregnant women, who were in the late stage of pregnancy, were diagnosed with COVID-19; one patient was admitted for fever with limb asthenia, and the other patient was admitted for abnormal chest computed tomography results.

**Diagnoses:**

Both pregnant women were diagnosed with COVID-19.

**Interventions:**

After the medical staff prepared for isolation and protection, the 2 pregnant women quickly underwent cesarean sections. A series of tests, such as laboratory, imaging, and SARS-CoV-2 nucleic acid examinations, were performed on the 2 women with COVID-19 and their newborns.

**Outcomes:**

One of the 2 infected pregnant women had severe COVID-19, and the other had mild disease. Both babies were delivered by cesarean section. Both of the women with COVID-19 worsened 3 to 6 days after delivery. Chest computed tomography suggested that the lesions due to SARS-CoV-2 infection increased. These women began to exhibit fever or reduced blood oxygen saturation again. One of the 2 newborns was born prematurely, and the other was born at full term. Neither infant was infected with COVID-19, but both had increased prothrombin time and fibrinogen, lactate dehydrogenase, phosphocreatine kinase, and creatine kinase isoenzyme contents.

**Lessons:**

SARS-CoV-2 infection was not found in the newborns born to the 2 pregnant women with COVID-19, but transient coagulation dysfunction and myocardial damage occurred in the 2 newborns. Effective management strategies for pregnant women with COVID-19 will help to control the outbreak of COVID-19 among pregnant women.

## 1 Introduction

In December 2019, a new virus, later named severe acute respiratory syndrome coronavirus 2 (SARS-CoV-2), was identified among pneumonia patients in Wuhan, a city in the Chinese province of Hubei.^[1]^ The disease caused by SARS-CoV-2, coronavirus disease 2019 (COVID-19), can cause serious and fatal respiratory illness but can also manifest with mild or no symptoms.^[2–5]^ The rapid global spread of SARS-CoV-2 in only 3 months highlights the transmissibility of this family of viruses and the significant morbidity and mortality that they can cause.^[6]^ However, another city in Hubei Province, Yichang city, with a resident population of 4.2 million people located 300 kilometers—only 2 hours by high-speed rail—from the center of the national epidemic in Wuhan, has reported 931 confirmed cases and 36 deaths from January 20, 2020, to April 9, 2020. The mortality rate was 3.87%, and a total of 894 patients were discharged from the hospital with a cure rate of 96.03%.^[7]^ Only 2 confirmed cases involved pregnant women during this period. At present, COVID-19 has spread throughout the world, and the number of infections is still rising. There are very few reports about pregnant women with COVID-19. The purpose of this study was to summarize infection prevention and control measures and to provide experience for the obstetric treatment and management of pregnant women with COVID-19 by reporting the maternal and neonatal outcomes of two pregnant women with COVID-19 in Yichang city, Hubei Province.

## 2 Materials and methods

### 2.1 Study design and patients

Due to the government's community investigation and mandatory hospitalization interventions, there were only 2 pregnant women with COVID-19 in Yichang city, which has a resident population of 4.2 million people, from January 20, 2020, to April 9, 2020. Both pregnant women spent the entire diagnosis and treatment process in the isolation ward of Yichang Central People's Hospital under the care of medical staff. We retrospectively reviewed the electronic medical records of the two pregnant women with COVID-19. The diagnosis of COVID-19 was based on the diagnostic criteria of the COVID-19 Prevention and Control Program (seventh edition) published by the National Health Commission of China.^[8]^

This study was reviewed and approved by the Clinical Research Ethics Committee of Yichang Central People's Hospital (Approval Number HEC-KYJJ2020-013-01). As this study is a retrospective case study, informed consent was waived by the ethical committee.

### 2.2 Data collection

This study analyzed the results of the 2 pregnant women's ages, pregnancy times, gestational weeks, clinical manifestations, laboratory test results, chest computed tomography (CT) results, SARS-CoV-2 nucleic acid detection results, treatments, neonatal birth conditions, and outcomes. In addition, this study followed up with the patients for one month after the mother and newborn recovered and were discharged.

### 2.3 Case presentations

#### 2.3.1 Case A

**Figure 1:**
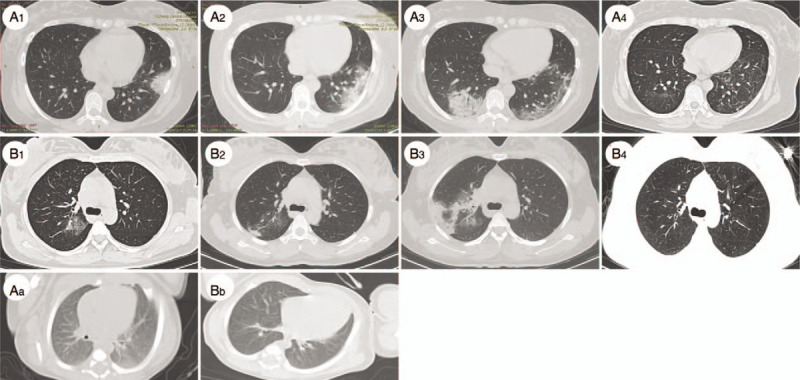
Chest CT (transverse plane) of 2 patients and their newborns. (A_1-4_) Patient A. A_1_: bilateral lung infection and ground-glass opacities of the left lower lobe on the day of cesarean section; A_2_: increased lesions 3 days after cesarean section; A_3_: lesions began to appear on the right side 3 days after cesarean section; A_4_: lesion absorption before discharge.(B_1-4_) Patient B. B_1_: bilateral lung infection and ground-glass opacities in the upper lobe of the right lung on the day of cesarean section; B_2_: lesions started to increase 3 days after cesarean section; B_3_: lesions increased further 3 days after cesarean section; B_4_: lesion absorption before discharge. A_a_: Newborn delivered by Patient A; B_b_: Newborn delivered by Patient B.

Patient A was a 33-year-old pregnant woman. Fifteen days before she was admitted to the hospital ward, her husband had returned to Yichang from Wuhan city. On February 7, 2020, she was 36^+3^ weeks pregnant and was admitted to the hospital because of “fever with asthenia for 2 days.” At the time of admission, the temperature of the pregnant woman was 38°C, and she exhibited limb asthenia. Her respiratory rate was 30 breaths/min, with 95% blood oxygen saturation, she occasionally experienced uterine contractions, and the fetal heart rate was 158 beats/min. Her cervical orifice in the vagina was dilated 50%, but the uterine cervical orifice was not opened, and the fetal head was at the ischial spine position S-2. The throat swab at the clinic was positive for SARS-CoV-2 nucleic acid. Chest CT showed bilateral lung infection and segmental consolidation of the left lower lobe (Fig. 1). According to the diagnostic criteria of the COVID-19 Prevention and Control Program,^[8]^ she was classified as a severe-type COVID-19 patient. Because of the severe COVID-19, a cesarean section was performed 3 hours after admission, and a boy weighing 2520 g was delivered. After the operation, breastfeeding initiation was delayed; anti-infection, uterine contraction-stimulating and anticoagulation therapeutics were given; and the pulmonary infection was treated with Arbidol (100 mg, 3 times daily), interferon (5 million U, twice daily), immunoglobulin (10 g daily), glucocorticoids (1–2 mg/kg/day), oxygen therapy (3 L/min), etc..^[8]^ On February 13, 3 days after the operation, the patient's blood oxygen saturation was 80% on room air. The chest CT infection foci intensified, and the patients’ medication dosages were increased and she was given chloroquine phosphate (500 mg twice daily) for the duration of her long hospitalization (Fig. 1). Three negative results of the mother's pharynx swab for the SARS-CoV-2 nucleic acid test were reported before she was discharged on March 17, 2020. The neonate's throat swab, anal swab, urine, and blood were negative for SARS-CoV-2. Congenital club foot was discovered in the neonate's physical examination. Myocardial injury was considered to be high by the detection of the myocardial enzyme spectrum index, and the neonate's condition improved with anti-infection treatment and myocardial nutrition. Five negative results of the neonate's pharyngeal swabs for SARS-CoV-2 nucleic acid tests were reported within 10 days before the mother and baby were discharged on March 17, 2020. The clinical details of mother A and her baby are shown in Table 1 and Table 2, respectively, and their chest CT images are shown in Figure 1.

**Table 1:**
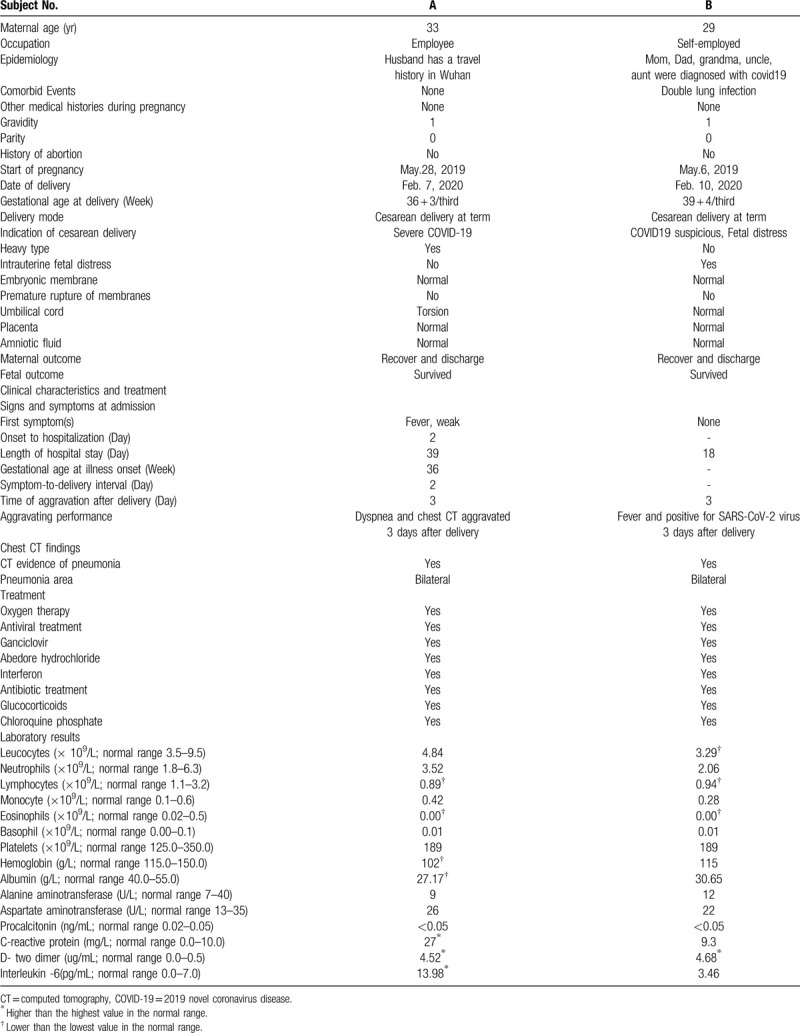
Maternal clinical and laboratory characteristics of mothers with COVID-19 infection.

**Table 2:**
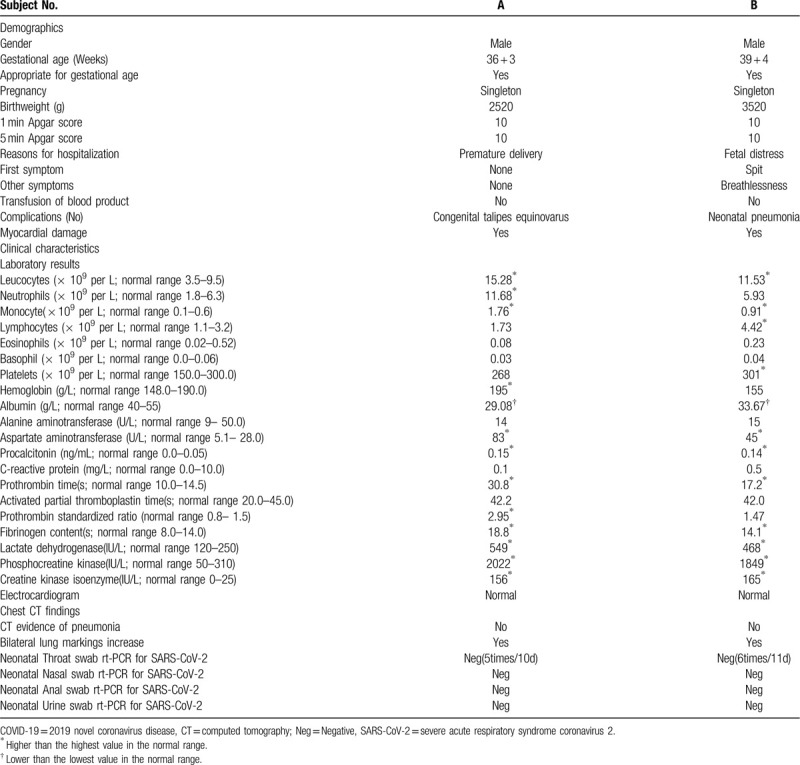
Information of neonates born to mothers with COVID-19 infection.

#### 2.3.2 Case B

Patient B was a 29-year-old pregnant woman, and her father, mother, grandmother, uncle, and aunt have all been diagnosed with COVID-19. On February 10, 2020, she was admitted to the hospital because her chest CT (Fig. 1) showed suspected COVID-19 during her labor examination at 39^+4^ weeks gestation. The throat swab at the clinic indicated that SARS-CoV-2 was suspected to be positive. At the time of admission, the pregnant woman had no fever, cough, abnormal fetal movement, or uterine contraction. Chest CT showed a bilateral lung infection and patchy high-density shadow in the upper lobe of the right lung. A cesarean section was performed 3 hours after admission on the same day because of fetal distress observed from the baseline fetal heart rate monitoring. A 3520 g boy was delivered, and a newborn isolation and observation period was conducted because of the baby's vomiting. On February 13, three days after the operation, the mother had a high fever of 39°C, and her throat swab for SARS-CoV-2 was positive. Moreover, her blood oxygen saturation dropped to 80% when breathing room air. The right lung foci on chest CT were enlarged, and the left lung foci were gradually absorbed (Fig. 1). The severity of her COVID-19 infection worsened from a suspected type to a confirmed severe type,^[8]^ so the treatment continued to be increased, and chloroquine phosphate (500 mg twice daily)^[8]^ was given. Three throat swabs for SARS-CoV-2 nucleic acid tests were negative, and the patient was discharged on February 28, 2020. The neonate's throat swab, anal swab, urine, and blood were also negative for SARS-CoV-2. However, the neonate also had a high myocardial enzyme spectrum index, indicating myocardial injury. Finally, the neonate improved through nutrition, anti-infection, and other treatments. Six of the neonate's pharyngeal swab tests for SARS-CoV-2 nucleic acid were negative within 11 days. The clinical details of mother B and her baby are shown in Table 1 and Table 2, respectively, and their chest CT images are presented in Figure 1.

## 3 Discussion

### 3.1 Outcomes and management of newborns born to pregnant women with COVID-19

We did not find SARS-CoV-2 infection in patients during the early and middle stages of pregnancy in all of Yichang city from January 20, 2020, to April 10, 2020, suggesting that patients in late pregnancy may be the susceptible population to respiratory diseases compared with patients in early and middle pregnancy.^[9–11]^ Both pregnant women with confirmed COVID-19 successfully delivered without death. Case A was the earlier confirmed to have COVID-19 at 36 weeks of gestation, but with more severe symptoms and longer hospital stays than case B. Both patients underwent a cesarean section immediately after admission because of ”severe COVID-19“ or ”fetal distress.” The 2 newborns were born without SARS-CoV-2 infection and were discharged from the hospital. Additionally, the outcomes of the newborns, even the preterm infant, were relatively good after delivery. It was suggested that labor should be induced in a timely manner when there was an obstetric indication or the condition of COVID-19 viral pneumonia in pregnant women was critical; thus, the development of severe or critical COVID-19 can be controlled as early as possible, which could also reduce the intrauterine infection of the fetus and the risk of neonatal asphyxia.^[12]^ Both cases of COVID-19 in parturient women were exacerbated 3 to 6 days after delivery. The infection foci on chest CT increased and their fever reappeared or their blood oxygen saturation decreased, suggesting that the stress of the cesarean section operation and the reduction in the lung function of pregnant women may make pregnant women with COVID-19, even those with COVID-19 pneumonia after delivery, progress very quickly to a more severe cytokine storm.^[13]^ Therefore, it is necessary to pay careful attention to the monitoring and treatment of pregnant women with COVID-19 after delivery. Moreover, both newborn patients exhibited high prothrombin time, fibrinogen content, lactate dehydrogenase levels, creatine phosphokinase levels, and creatine kinase isoenzyme levels as detected in the laboratory, which indicated that the case B newborn had transient coagulation functional and myocardial damage,^[14]^ but the electrocardiogram was normal, so it was possible that the newborn's transient hypoxia was caused by the decline in the maternal pulmonary function. Based on very limited case reports, we are cautiously optimistic about the impact of maternal COVID-19 on pregnancy outcomes, especially on the long-term prognosis of surviving newborns.

### 3.2 Is there a risk of SARS-CoV-2 vertical transmission through the placenta from mother to child?

The newborns of the 2 reported pregnant women with COVID-19 (even the severe type) were not infected with SARS-CoV-2 within 1 month after delivery. In addition to the virus being transmitted through droplets, direct contact, and the oral-fecal route, a recent study of 6 SARS-CoV-2-positive pregnant women found that SARS-CoV-2 antibodies can be detected in newborns.^[15]^ This study is based on the results of serum antibody testing for SARS-CoV-2 in accordance with the seventh edition of the New coronavirus pneumonia diagnosis and treatment guidelines^[8]^ and the results of serum antibody testing of newborns of infected pregnant women. All the pregnant women had mild COVID-19 and underwent a cesarean section in negative pressure isolation operating rooms during the third trimester. All the newborns were born in good condition. Pharyngeal swabs and blood samples collected and tested by real-time reverse transcription-polymerase chain reaction for the virus were negative. Both IgM and IgG were positive in the newborns; although there were only 6 cases, IgG was increased in 5 infants. It should be noted that two of the infants had higher IgM and IgG levels than normal infants, and their mothers had antibody levels that were higher than normal. Maternal IgG can be passed to the baby through the placenta, but IgM cannot be transmitted vertically through the placenta. However, it is still uncertain whether the increase in IgM in newborns is due to placental destruction or the transmission of the maternal virus from the uterus to the fetus. Another case^[16]^ of IgM detected in a neonate infected by a pregnant women was reported, which complements the above 6 cases. The study also tested vaginal swabs from the infected woman, which were negative. The newborn had a birth weight of 3120 g and had no clinical symptoms. The newborn's nasopharyngeal swabs were negative until 16 days after delivery. However, the serum levels of SARS-CoV-2 IgG and IgM in the newborn increased and declined slowly, possibly due to placental damage or virus transmission. There is no evidence to support the risk of vertical transmission of the SARS-CoV-2 from mother to child through the placenta. Possibly due to the limited sample size and the effect of the gestational week of the patient, no SARS-CoV-2 infection in late pregnancy caused vertical transmission and intrauterine infection in the fetus.

### 3.3 Perioperative management strategies for pregnant women with COVID-19

Expert advice on the new coronavirus infection during pregnancy and puerperium suggests that COVID-19 is not an indication for the termination of pregnancy.^[12]^ The indication for the termination of pregnancy depends on the disease condition, gestational age, and fetal condition of the pregnant woman. Sixteen COVID-19 clinically confirmed pregnant women terminated their pregnancy due to ”scarred uterus, premature rupture of membranes, respiratory pneumonia, or sociopsychological factors requiring termination of pregnancy".^[17]^ However, a 3670 g female newborn was delivered via vaginal delivery by a 30-year-old COVID-19-positive pregnant woman due to maternal request without adverse outcomes for the mother, infant, or healthcare staff.^[18]^ When evaluating the condition of a pregnant woman and requiring an emergency cesarean section, the operating room staff should be informed in advance to receive the pregnant woman in the negative pressure operating room, and level-three protection of the whole staff should also be used. Anesthesiologists and newborn pediatricians should arrive at the operating room in advance and actively prepare the recovery platform, rescue drugs, instruments, etc. The first method of anesthesia is intraspinal anesthesia. Anesthesiologists should wear positive pressure breathing headgear and avoid unnecessary suction when removing the catheter because the bodily secretions of the parturient can be aerosolized. The umbilical cord was cut immediately during the operation to reduce the mother's blood contamination of the newborn's blood. The SARS-CoV-2 infection precautions should be maintained from the isolated hallway to the neonatal ICU isolation treatment room. The vertical transmission risk of the new coronavirus should be eliminated. The newborns were examined for blood biochemistry, influenza, and respiratory tract-related pathogens and were given infusion support therapy or artificial feeding. During hospitalization, the mother continued lactation unobstructed but stopped breastfeeding. After being cured and discharged from the hospital, the pair were isolated for 14 days and reexamined without abnormal mother-infant contact or breastfeeding.

### 3.4 How can pregnant women be prevented from spreading the virus in the community during the COVID-19 outbreak?

Pregnant women are at a high risk of contracting SARS-CoV-2 infection, but there are no effective drugs or vaccines against the disease.^[19]^ Therefore, strict personal protection must be taken.^[20,21]^ Isolation at home of confirmed or suspected patients with mild illness is recommended. Reducing the number of regular prenatal visits, taking pregnancy education courses through the Internet and participating in online pregnancy consultation and medical services are options for pregnant women to reduce their risk of infection. Residences should have adequate ventilation with sunlight to allow for the destruction of SARS-CoV-2. People have been asked to wear masks in public, and crowded places and large-scale gatherings (entertainment parks, etc.) have been prohibited. Only 2 cases of pregnant women infected with COVID-19 were reported in Yichang city from January 20, 2020, to April 9, 2020, mainly because of the comprehensive, strict and thorough prevention and control measures (such as encouraging the public to wash their hands frequently, wear masks and keep social distance; carrying out large-scale temperature monitoring; suspending public gatherings and calling on the public to reduce travel) that were taken by the government in response to the epidemic.

Our cases provide preliminary results of the effective prevention and control of COVID-19 in pregnant women and the potential risks of vertical transmission. However, due to the small sample size and the limitations of gestational age, a large number of comprehensive data on pregnant women with COVID-19 are still needed to better understand the impact of COVID-19 on maternal and infant outcomes.

## Author contributions

**Conceptualization:** Tingting Zheng.

**Data curation:** Tingting Zheng.

**Formal analysis:** Jianqiang Guo.

**Investigation:** Jianqiang Guo.

**Project administration:** Hong Ye.

**Resources:** Wencong He, Hao Wang, Huiling Yu.

**Writing – original draft:** Tingting Zheng.

**Writing – review & editing:** Tingting Zheng, Jianqiang Guo, Hong Ye.
